# Reduction of mutant huntingtin accumulation and toxicity by lysosomal cathepsins D and B in neurons

**DOI:** 10.1186/1750-1326-6-37

**Published:** 2011-06-01

**Authors:** Qiuli Liang, Xiaosen Ouyang, Lonnie Schneider, Jianhua Zhang

**Affiliations:** 1 Department of Pathology, Center for Free Radical Biology, University of Alabama at Birmingham, Birmingham, USA; 2 Center for Free Radical Biology, University of Alabama at Birmingham, Birmingham, USA; 3 Department Veterans Affairs, Birmingham VA Medical Center, University of Alabama at Birmingham, Birmingham, USA

**Keywords:** huntingtin, lysosome, cathepsin, autophagy

## Abstract

**Background:**

Huntington's disease is caused by aggregation of mutant huntingtin (mHtt) protein containing more than a 36 polyQ repeat. Upregulation of macroautophagy was suggested as a neuroprotective strategy to degrade mutant huntingtin. However, macroautophagy initiation has been shown to be highly efficient in neurons whereas lysosomal activities are rate limiting. The role of the lysosomal and other proteases in Huntington is not clear. Some studies suggest that certain protease activities may contribute to toxicity whereas others are consistent with protection. These discrepancies may be due to a number of mechanisms including distinct effects of the specific intermediate digestion products of mutant huntingtin generated by different proteases. These observations suggested a critical need to investigate the consequence of upregulation of individual lysosomal enzyme in mutant huntingtin accumulation and toxicity.

**Results:**

In this study, we used molecular approaches to enhance lysosomal protease activities and examined their effects on mutant huntingtin level and toxicity. We found that enhanced expression of lysosomal cathepsins D and B resulted in their increased enzymatic activities and reduced both full-length and fragmented huntingtin in transfected HEK cells. Furthermore, enhanced expression of cathepsin D or B protected against mutant huntingtin toxicity in primary neurons, and their neuroprotection is dependent on macroautophagy.

**Conclusions:**

These observations demonstrate a neuroprotective effect of enhancing lysosomal cathepsins in reducing mutant huntingtin level and toxicity in transfected cells. They highlight the potential importance of neuroprotection mediated by cathepsin D or B through macroautophagy.

## Background

A common feature of neurodegenerative diseases, including Alzheimer's, Parkinson's and Huntington's diseases, is the accumulation of aggregation-prone proteins, such as β-amyloid in Alzheimer's disease, α-synuclein in Parkinson's disease and mutant huntingtin (mHtt) in Huntington's disease [1]. It is generally thought that the response of the neuronal cell to these aggregated proteins determines whether cell death or dysfunction occurs [1]. In this respect the autophagy-lysosomal pathway is particularly important. Lysosomal-mediated macroautophagy is largely responsible for degradation of intracellular damaged or aggregated proteins. The macroautophagy process involves formation of autophagosomes, transportation of damaged or aggregated proteins to the lysosomes, and degradation of these proteins by lysosomal proteases. Because of this capability for high capacity protein degradation inherent in macroautophagy the pathway has been identified as a potential target for the removal of mHtt protein. Previous studies have explored the potential of up-regulating autophagosomal formation by rapamycin, trehalose and lithium, and this resulted in the decreased mHtt aggregation and toxicity *in vitro *[2,3]. Recent studies in the context of Alzheimer's disease models have indicated that macroautophagy is a highly efficient process in neurons, and the activities of lysosomal proteins are rate limiting in degrading aggregated proteins [4]. If lysosomal activities are rate limiting, enhancing their activities may alleviate the burden to the proteasomes that are also involved in degradation of huntingtin [5,6]. Supporting this notion, dysfunction in the lysosomal pathway has long been implicated in aging and neurodegenerative diseases [7-17]. Thus, investigating the impact of enhancing lysosomal proteins on mutant huntingtin accumulation and toxicity is of particular importance.

Lysosomal proteases that are highly expressed in the brain include the aspartate protease Cathepsin D (CathD) and the cysteine protease (CathB) [7-17]. Loss of cathepsins in processing damaged or aggregated proteins has been demonstrated in neurological disorders as well as mouse neurological disease models [7,18-20]. For example, deficiency of CathB has been shown previously to exacerbate Aβ accumulation in a mouse model for Alzheimer's disease and overexpression of CathB has been shown to reduce Aβ load [18]. In addition, we and others have previously shown that mice with deficient lysosomal CathD exhibited significant α-synuclein accumulation in their brains, indicating a critical role for CathD in mediating α-synuclein metabolism [19,20]. This is important because α-synuclein mutation and gene amplification is responsible for a small subset of familial Parkinson's disease cases, and α-synuclein is a major component of Lewy bodies in a majority of sporadic Parkinson's disease patients [21]. *In vitro*, we have shown that overexpression of CathD decreases the level of α-synuclein aggregation and protects against α-synuclein-mediated toxicity [19,20]. Similarly, in Parkinson's disease research, proteolytic reduction of aggregation-prone and neurotoxic mutant huntingtin is important in Huntington's disease research. Because the huntingtin gene is essential for development [22], the simple reduction of the huntingtin gene may not be ideal therapeutic strategy. Allelic reduction of mutant huntingtin gene can prevent further production of the product, but lacks the potential to clear accumulated huntingtin toxic protein products. Determining which proteases are neuroprotective and which are detrimental to neurons is critically needed, considering the existence of proteasomes and distinct families of proteases in the cell that are capable of digesting mutant huntingtin to a varying extent [5,6].

In the present study, we investigated whether enhancing individual lysosomal proteases may be effective in reducing mHtt using a range of molecular approaches. Because full-length mHtt is produced in Huntington's disease patients and may be more relevant to neuron cell death mechanisms [23], we investigated the effects of lysosomal enzymes on the toxicity of the full-length mHtt in neurons. Our finding indicated that lysosomal CathD and CathB reduced mHtt level, and protected against mHtt toxicity in primary neurons. Furthermore, CathD and CathB neuroprotective effects are dependent on autophagy.

## Methods

### Reagents and antibodies

3-methyladenine (3-MA), pepstatin A (PepA) and E64d were purchased from Sigma-Aldrich (St. Louis, MO, USA). Nucleofector Kit was from Lonza (Walkersville, MD, USA). Lipofectamine 2000 was from Invitrogen.

Anti-huntingtin monoclonal antibody 2166 (Ab2166), anti-polyglutamine expansion monoclonal antibody MAB1574 (clone 1C2) and anti-huntingtin monoclonal antibody (clone mEM48) were purchased from Millipore (Temecula, CA, USA). Anti-cathepsin D and Anti-cathepsin B antibodies were obtained from Santa Cruz Biotechnology (Santa Cruz, CA, USA). Anti-LAMP1 was from Novus Biologicals (Littleton CO, USA). Anti-LC3 antibody was from Sigma-Aldrich (St. Louis, MO, USA). Anti-β-actin monoclonal antibody was from Sigma-Aldrich. Fluorescent Alexa 488 (anti-mouse), fluorescent Alexa 568 (anti-goat) and fluorescent Alexa 488 (anti-rabbit) secondary antibodies were from Invitrogen (Carlsbad, CA, USA). Horseradish peroxidase-labeled secondary antibodies for enhanced chemiluminescence system detection were from Pierce (Rockford, IL USA).

### Plasmids

Full-length Htt with 23 or 145 polyQ repeats using pcDNA vector were purchased from Invitrogen. Plasmids encoding human cathepsin D and B in pCMV-5a expression vectors were purchased from Origene.

### HEK293 cell culture and transfection

HEK293 cells were grown in Dulbecco's modified Eagle's medium (DMEM) (Invitrogen) supplemented with 10% fetal bovine serum (FBS) (Atlanta Biologicals), and 100 U/ml penicillin/streptomycin (Invitrogen) at 37°C, and 5% CO_2_. Transfections were performed using lipofectamine 2000 according to the manufacturer's instructions. Cell death was measured by trypan blue exclusion, MTS (3-(4,5-dimethylthiazol-2-yl)-5-(3-carboxymethoxyphenyl)-2-(4-sulfophenyl)-2H-tetrazolium) colorimetric and Calcein AM viability assays.

### Primary neuron cultures and transfection

The animal studies have been approved by the University of Alabama at Birmingham IACUC. Primary cortical neurons were obtained from embryonic day 18 (E18) embryos. Timed-pregnant Sprague-Dawley rats (Charles River Laboratories, Wilmington, MA, USA) were sacrificed by CO_2 _inhalation and embryos were collected in a Petri dish and placed on ice. Dissections were performed in ice cold Hanks' balanced sodium salts (without Ca^2+ ^and Mg^2+^). Cerebral cortices were isolated and collected in a 15 ml Falcon tube. The tissues were incubated for 15 min at 37°C with papain (Worthington). The tissues were mechanically dissociated with a fire-polished Pasteur pipette. Cells were finally concentrated by centrifugation at 25°C for 5 min at 1000 × g and resuspended in Neurobasal medium containing 2% B27 supplement (Invitrogen), 1% Pen-Strep (10,000 U/ml, 10,000 μg/ml) and 0.5 mM L-glutamine. Electroporation was performed using Rat Nucleofector Kit with Nucleofector II machine. Cells were then plated in multiwell 24-well or 6-well plates coated with 0.1 mg/ml Poly-L-Lysine (Sigma). The cultures were kept in a humid incubator (5% CO_2_, 37°C) and half of the medium was changed once a week.

### Immunocytochemistry

Cells on coverslips were washed by room temperature PBS and fixed in 4% paraformaldehyde for 45 min. After washing with PBS, the fixed cells were then permeabilized with PBS-T (0.1% Triton X-100 in PBS) for 3 min. The cells on coverslips were blocked in PBS supplemented with 3% BSA for 1 hour at room temperature. The cells were then incubated with a primary antibody in blocking solution overnight at 4°C and then washed 3 times with PBS. The secondary antibody conjugated to fluorophores was incubated for 1 hr at room temperature. Before mounting in fluoromount G on microscope slides, coverslips were incubated in 2 μg/ml Hoechst 33342 to label nuclear DNA. Stained cells were visualized by a fluorescent microscope (Leica Microsystems, Wetzlar, Germany). Staining was visualized on a Zeiss Axiocam CCD camera on a 100 W Axioscope bright field and fluorescence microscope.

### Measurement of neuronal death

For the cell death assay, primary cortical neurons were electroporated the day of plating with the rat neuron nucleofector kit (Lonza) and then plated in 24-well plates with coverslips. At 9 DIV (day *in vitro*), cells were fixed and immunostained with mAb2166 antibody as above, and nuclei counter-stained by Hoechst to identify degenerated neurons by counting mAb2166-positive neurons with nuclei shrinkage or fragmentation. We selected 15-20 random fields for each coverslip to take images for quantifying cell death. Each graph represents three independent experiments. Data are expressed as the percentage of neuronal cell death.

### Western blot analysis

Total cellular extracts were collected in lysis buffer containing 50 mM Tris-HCl pH7.4, 150 mM NaCl, 5 mM EDTA, 1% Triton X-100 and supplemented with protease inhibitor mixture (Roche). Homogenates were centrifuged at 10,000 × g for 15 min at 4°C. Protein concentrations were determined by detergent-compatible protein assay (Bio-Rad). Protein extracts were mixed with 5 × sample loading buffer and boiled for 5 min. Thirty to fifty micrograms of protein was resolved on 12% SDS-PAGE gel and transferred to nitrocellulose membranes. Membranes were blocked in 5% non-fat dry milk or 5% horse serum (for cathepsin D antibody detection) in TBST (50 mM Tris-HCl, 150 mM NaCl, pH7.4, 0.1% Tween 20) for 30 min at room temperature. The membranes were then incubated overnight at 4°C with primary antibodies: anti-mAb2166 1:1000; anti-1C2 1:5000; anti-EM48 1:1000; anti-cathepsin D 1:1000; anti-cathepsin B 1:1000; anti-LC3 1:1000 or anti-actin 1:5000. The membranes were then washed 4 times with TBST and incubated with horseradish peroxidase-conjugated secondary antibody for 1 h at room temperature. After washing for 40 min with TBST, the membranes were developed using enhanced chemiluminescence (ECL) substrate kit. We used U-Scan-IT software to quantify the western blot band intensity.

### CathD and CathB activity assay

CathD activity was measured using an assay kit from Sigma. Cells were lysed in 20 mM MES pH6.8 containing 80 mM NaCl, 1 mM MgCl_2_, 2 mM EGTA, 10 mM NaH_2_PO4, proteinase inhibitor cocktail and phosphotase inhibitor cocktail. Ten μg of cell lysate were assayed in a 96-well plate according to protocol described by the manufacturer. CathB activity was measured using a kit from Biovision. Cells were lysed with the cell lysis buffer supplied with the kit. CathB activity was measured in a 96-well plate according to manufacturer's instruction.

### Real-time RT-PCR

Total RNA was extracted using TRIZOL reagent from Invitrogen. cDNA was synthesized using an iScript cDNA synthesis kit(BioRad) following the manufacturer's instructions.

Real-time PCR was performed to determine the mRNA levels of Htt and CathD and CathB. Primer sequences were: GAPDH, 5'- GCCAAAAGGGTCATCATCTC-3' and 5'GGCCATCCACAGTCTTCT-3'; CathD, 5'-TTCCCGAGGTGCTCAAGAACTACA -3'and 5'-TGTCGAAGACGACTGTGAAGCACT -3'; CathB, 5'-AAGCTTCGATGCACGGGAACAA-3' and 5'- ATGCAGATCCGGTCAGAGAT-3'.

Htt, 5'- CTCATTTCTCCGTCAGCACA-3' and 5'- CAAAGCTTCACAGCATCCAA-3'.

The mRNA level of GAPDH served as an internal control.

### Statistical analysis

Data are reported as means ± SEM. Comparisons between two groups were performed with unpaired Student's *t-*tests. Comparisons among multiple groups or between two groups at multiple time-points were performed by either one-way or two-way analysis of variance, as appropriate. A *p *value of less than 0.05 was considered statistically significant.

## Results

### Overexpression of cathepsins D (CathD) and B (CathB) reduce mHtt level in human embryonic kidney (HEK) cells

**Figure 1:**
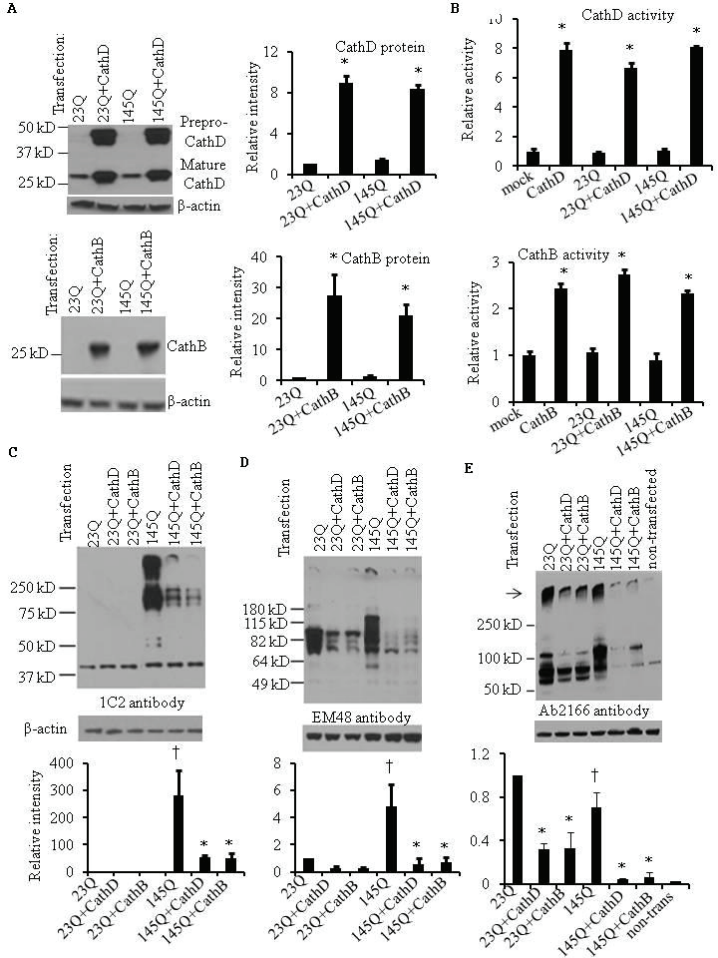
**Cathepsin D (CathD) and B (CathB) reduce Htt loads in HEK cells**. HEK cells transfected by lipofectamine with different Htt and cathepsin cDNA constructs were harvested 48 hr later for real-time quantitative RT-PCR, western blot and enzymatic activity analyses. **A**. Transfection of cathepsin D (CathD) and B (CathB) increases their expression in HEK cells. HEK cells were transfected with 23QHtt, 23QHtt plus CathD, 23QHtt plus CathB, 145QmHtt, 145QmHtt plus CathD, and 145QmHtt plus CathB constructs for 48 hr. Western blot analyses were performed with anti-CathD and anti-CathB antibodies. β-actin western blots were used as loading controls. Pre-pro forms of CathD are 49 and 50 kD. Mature CathD is 32 kD. Mature CathB is 27 kD. Relative expression levels were quantified by band intensity. Student t-test was performed. *p < 0.05 compared to without cathepsin transfection. **B**. Increase of CathD and CathB enzymatic activities after transfection. HEK cells were transfected with CathD and CathB, and as described in A. CathD and CathB activities were assayed by CathD or CathB activity assay kit. *p < 0.05 compared to without cathepsin transfection. **(C-E) **Transfection of CathD and CathB reduce transfected Htt levels. HEK cells were transfected with 23QHtt, 23QHtt plus CathD, 23QHtt plus CathB, 145QmHtt, 145QmHtt plus CathD, and 145QmHtt plus CathB constructs. Western blot analyses were performed first with (**C**) 1C2 antibody that is specific for the polyQ of 145QmHtt, and then with (**D**) EM48 that preferentially recognizes the aggregates or (**E**) Ab2166 antibody that recognizes both 23Q and 145QHtt. β-actin western blots were used as loading controls. *p < 0.05 compared to without cathepsin transfection. †*p *< 0.05 compared to 23Q transfection.

Consistent with prior studies [24], we found that overexpressing full-length Htt protein with a short polyQ repeat (23QHtt) or mHtt protein with a long polyQ repeat (145QmHtt) did not induce cell death assessed by three independent methods (Calcein AM, MTS (3-(4,5-dimethylthiazol-2-yl)-5-(3-carboxymethoxyphenyl)-2-(4-sulfophenyl)-2H-tetrazolium) colorimetric cell survival, or trypan blue exclusion assays). Cells transfected with full-length 23QHtt and 145QmHtt were used to investigate whether enhancing lysosomal cathepsin level can reduce Htt or mHtt protein levels. For this purpose, we co-transfected HEK cells with plasmids encoding lysosomal CathD or CathB, together with plasmids encoding full-length Htt or mHtt proteins. Western blot analyses show that the expression of both cathepsin precursors and mature proteins are significantly increased 8-25 fold, regardless of whether the cells contained 23QHtt or 145QmHtt (Figure 1A). These data indicate that mHtt did not affect CathD or CathB processing to the mature forms. By western blot analyses, we found no significant compensation or cross regulation of CathB as a consequence of overexpressing CathD and vice versa (Additional file 1, Figure S1A). Real-time RT-PCR results showed that the mRNA levels of CathD or CathB are greatly increased in CathD or CathB transfected cells with or without 23QHtt or 145QmHtt (Additional file 1, Figure S1B). In addition to the increases of CathD or CathB protein and mRNA levels, we found significant increase of enzymatic activities in CathD or CathB transfected cells with or without 23QHtt or 145QmHtt (Figure 1B). CathD or CathB overexpression did not cause an off-target degradation of proteins, as indicated by western blot analyses of mitochondrial outer membrane protein VDAC and endoplasmic reticulum protein calnexin (Additional file 1, Figure S1C). To determine how overexpression of cathepsins affect the total level and cleaved Htt, we performed western blot analyses using 1C2 antibody that is specific for the polyQ of 145QmHtt (Figure 1C), EM48 that preferentially recognizes the aggregates (Figure 1D) and Ab2166 that recognizes both Htt and mHtt (Figure 1E). We found that CathD and CathB significantly reduced both full-length and cleaved forms of Htt and mHtt as detected by all 3 antibodies (Figure 1C-1E). The species of 23QHtt and 145QmHtt recognized by these antibodies are similarly decreased by CathB and CathD. Endogenous Htt levels are not significantly decreased by CathD or CathB transfection (Additional file 1, Figure S1D), suggesting that CathD or CathB has more effect on decreasing excessive exogenous htt levels. The RNA levels of Htt were not affected by CathD or CathB transfection as shown by quantitative RT-PCR (Additional file 1, Figure S1E), suggesting that all the transfections had similar transfection efficiency.

### Cathepsin D (CathD) and B (CathB) inhibitors exacerbate mHtt toxicity in primary neurons

**Figure 2:**
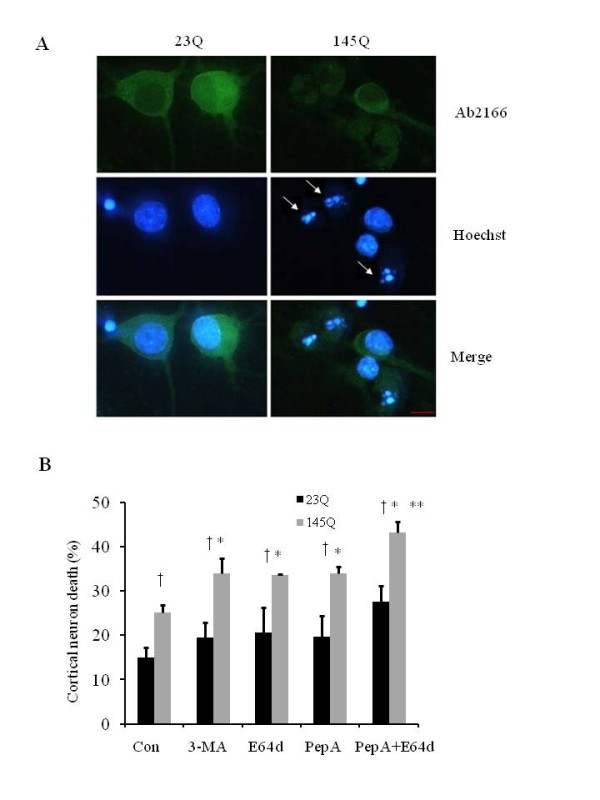
**Cathepsin D (CathD) and B (CathB) inhibitors exacerbate 145QmHtt-induced cell death in cortical neurons**. **A**. 145QmHtt transfected neurons exhibited increased cell death. Primary cortical neurons were transfected with 23QHtt and 145QmHtt constructs. At 9 DIV, neurons were immunostained by anti-Htt Ab2166 and nuclei were stained by Hoechst. Arrows point to the pyknotic nuclei. Scale bar = 10 micron. **B**. 3-MA, pepstatin A and E64d exacerbated 145QmHtt-induced neuron death. The combined use of pepA and E64d further exacerbated 145Q-mhtt-induced neuron death. Primary cortical neurons (9 DIV) were treated with 3-MA (10mM), CathD and B inhibitors, pepstatin A (15 μM) and E64d (30 μM), respectively, for 24 hr. Con = empty vector control. Neuronal cell death was scored by counting pyknotic nuclei after Hoescht staining. There is no statistic significant difference in 23QHtt transfected neurons in the absence or presence of any of the chemicals. *p < 0.05 Student t-test compared to no inhibitor. †*p *< 0.05 compared to 23Q transfection. **p < 0.05 Student t-test compared to either pepA or E64d alone.

Huntington's disease patients exhibit neurodegeneration in both cortex and striatum [25]. Because we did not find a significant increase of cell death after 145QmHtt transfection compared to 23QHtt transfection in HEK cells, we investigated the effects of 145QmHtt versus 23QHtt on cell death in primary cortical neurons. We harvested primary cortical neurons from embryonic day 18 rats, transfected with full-length 23QHtt and 145QmHtt, and cultured *in vitro *for 9 days (9 DIV). Transfection efficiency was consistently ~30% of all neurons, as detected both by transfection with control plasmid encoding GFP protein, and by co-transfection of GFP and 23QHtt or 145QmHtt constructs. We also stained the cells with Ab2166 which recognizes both 23QHtt and 145QmHtt proteins, and confirmed the transfection efficiencies with these plasmids encoding 23QHtt or 145QmHtt. To determine mHtt-induced cell death, we stained the neuron cultures with Ab2166 antibody and counter-stained with Hoechst for nuclei. Dying neurons exhibited nuclei with a pyknotic morphology (Figure 2A). We found that 145QmHtt induced significantly more cell death than 23QHtt in these neurons (Figure 2B). To study the effects of inhibiting the lysosomal pathway on 145QmHtt-induced cell death, we treated the 23QHtt and the 145QmHtt-transfected neurons with CathD and B inhibitors, pepstatin A (pepA) and E64d, respectively. We found that these inhibitors exacerbated 145QmHtt-induced neuronal cell death. In addition, the PI3K inhibitor 3-MA, which inhibits autophagosomal formation, increased toxicity to a similar extent as that of the cathepsin inhibitors in the presence of 145QmHtt, whereas it had no effect on cell death in the presence of 23QHtt (Figure 2B). The combined use of pepA and E64d further exacerbated 145Q-mHtt-induced neuron death compared to either inhibitor alone (Figure 2B).

### Overexpression of CathD and CathB reduce mHtt neurotoxicity in primary neurons

**Figure 3:**
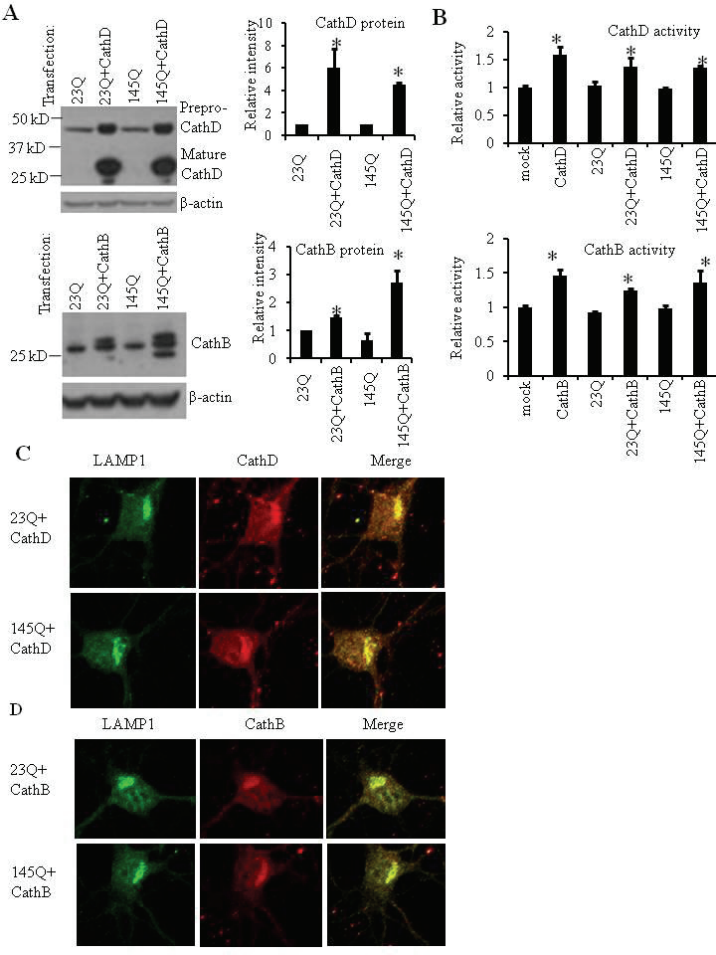
**Cathepsin D (CathD) and B (CathB) expression and localization in primary neurons**. **A**. Western blot analyses of CathD and CathB protein levels after transfection. Primary cortical neurons were transfected with 23QHtt, 23QHtt plus CathD, 23QHtt plus CathB, 145QmHtt, 145QmHtt plus CathD and 145QmHtt plus CathB constructs. Western blot analyses were performed with anti-CathD and anti-CathB antibodies. β-actin western blots were used as loading controls. Relative expression levels were quantified by band intensity. Positions of molecular weight markers were indicated. Quantification of the western blots are shown in the bar graphs. *p < 0.05 compared to without cathepsin transfection. **B**. Analyses of CathD and CathB enzymatic activities after transfection. Primary cortical neurons were transfected with CathD, CathB and as described in A. CathD and CathB activities were assayed by CathD or CathB activity assay kit. *p < 0.05 compared to without cathepsin transfection. **C and D**. Colocalization of transfected CathD (**C**) and CathB (**D**) with LAMP-1. CathD and CathB transfected neurons were examined by co-immunostaining of LAMP-1 and CathD or CathB. Yellow colored cytoplasmic spots are indicative of co-localization of transfected cathepsins and LAMP-1. Scale bar = 10 micron.

We next examined whether increasing lysosomal activities reduces mHtt toxicity in primary cortical neurons. With ~30% transfection efficiency, we found that the protein expression levels of CathD and CathB are increased by transfection of plasmids encoding CathD and CathB, in primary cortical neurons (Figure 3A). Quantification of the western blots indicated that the increase of CathD and CathB are between 0.5 and 5 fold (Figure 3A). Real-time PCR results showed that mRNA levels of CathD or CathB are greatly increased in CathD or CathB transfected cells with or without 23QHtt or 145QmHtt (Additional file 2, Figure S2). In addition to increases in CathD or CathB protein and mRNA levels, we found significant increase of enzymatic activities in CathD or CathB transfected cells with or without 23QHtt or 145QmHtt (Figure 3B). We found that all the CathD and CathB colocalized to the lysosomes, as indicated by the co-immunostaining of CathD or CathB with LAMP1 (Figure 3C and 3D).

**Figure 4:**
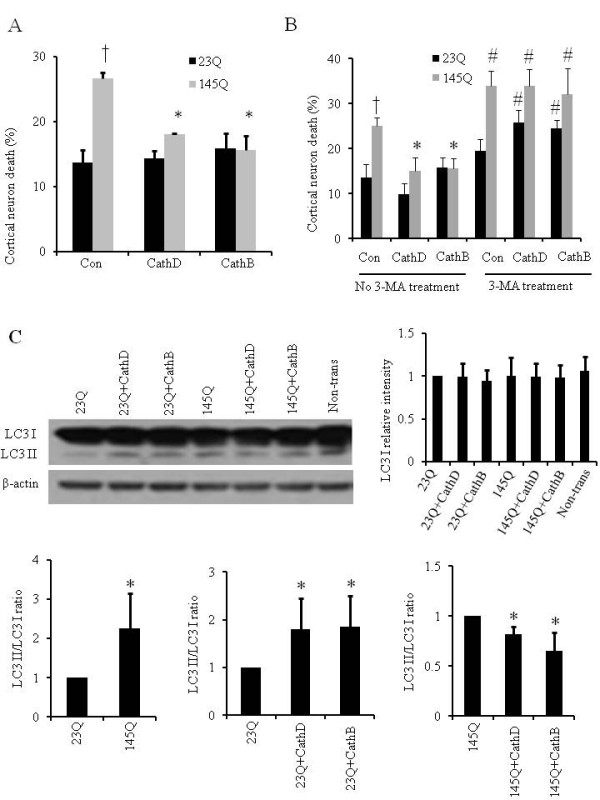
**Cathepsin D (CathD) and B (CathB) reduce mHtt-induced toxicity in primary cortical neurons**. **A**. Neurons were transfected with full length 23QHtt or 145QmHtt, either alone or co-transfected with CathD or CathB. Con = empty vector control. Cell death was increased by 145QmHtt and reduced by co-transfection with CathD or CathB. *p < 0.05 compared between 145QmHtt alone versus 145QmHtt co-transfection with CathD or CathB. †*p *< 0.05 compared between 145QmHtt and 23QHtt transfected cells. **B**. Blockade of autophagy reduces the effects of CathD and CathB in neuroprotection against 145QmHtt induced cortical neuron death. In 23QHtt transfected neurons, overexpression of CathD or CathB, or 3-MA inhibition alone did not cause neuron death. However, in these 23QHtt transfected neurons when autophagy is blocked by 3-MA, increasing CathD or CathB increased cell death. In 145QmHtt transfected neurons, CathD and CathB reduced 145QmHtt-induced neuron death. When autophagy is blocked by 3-MA, 145QmHtt is more toxic, and CathD or CathB enhancement could no longer reduce 145QmHtt-induced cell death. Con = empty vector control. †*p *< 0.05 compared between 23QHtt transfected versus 145QmHtt transfected cells. *p < 0.05 compared between 145Q and 145Q+CathD or CathB transfected cells. #*p *< 0.05 compared between transfected cells with 3-MA treatment and without 3-MA treatment. **C**. 145QmHtt increases LC3 II/LC3 I ratio. Primary neurons were transfected with 23QHtt, 23QHtt plus CathD, 23QHtt plus CathB, 145QmHtt, 145QmHtt plus CathD, and 145QmHtt plus CathB constructs. Western blot analyses were performed with an anti-LC3 antibody. β-actin western blots were used as loading controls. Immunoreactive bands were quantified and shown in the graphs. *p < 0.05 compared between 145QmHtt and 23QHtt transfection.

Under these conditions, we found that 145QmHtt is significantly more toxic than 23QHtt (Figure 4A), and that 145QmHtt toxicity was reduced by co-transfection with either CathD or CathB (Figure 4A). To determine whether CathD and CathB neuroprotection against mHtt toxicity is through an autophagy-mediated mechanism, we investigated whether blockade of autophagy reduces the neuroprotective effects of CathD and CathB against 145QmHtt toxicity in primary cortical neurons. We used 3-MA as an inhibitor for the autophagy pathway. In 23QHtt transfected neurons, overexpression of CathD or CathB, or 3-MA inhibition alone did not cause neuron death. However, in these 23QHtt transfected neurons when autophagy is blocked by 3-MA, increasing CathD or CathB increased cell death (Figure 4B). In 145QmHtt transfected neurons, CathD and CathB reduced 145QmHtt-induced neuron death (Figure 4B). When autophagy is blocked by 3-MA, 145QmHtt is more toxic, and CathD or CathB enhancement could no longer reduce 145QmHtt-induced cell death (Figure 4B). Consistent with prior studies in mHtt knock-in mice that autophagic stress is induced by mHtt [26], we found that the ratio of LC3 II/LC3 I was increased significantly in 145QmHtt transfected neurons compared to 23QHtt transfected neurons (Figure 4C). Western blot analyses showed that in both 23QHtt and 145QmHtt transfected neurons, co-transfection of either CathD or CathB changed the LC3II/LC3 I ratio, suggesting that CathD and CathB changes the autophagy dynamics in response to the overexpression of either wildtype or mutant Htt protein (Figure 4C). In the absence of Htt, CathD or CathB did not significantly change the ratio of LC3 II/LC3 I (Additional file 1, Figure S3).

## Discussion

Understanding the mechanisms of clearance of toxic mutant huntingtin is essential in order to explore therapeutic strategies against Huntington's disease. Here we examined the role of enhancing lysosomal CathB or D regarding the consequence of whether they are detrimental or protective against mutant huntingtin toxicity. We found that overexpression of full-length Htt with 23 polyQ (23QHtt) and mHtt with 145 polyQ (145QmHtt) had no effects on cell viability in HEK cells. This allowed an assessment of the ability of CathB and D to process huntingtin protein in the absence of the confounding effects of cell death. Prior studies indicated that CathD is more effective in reducing α-synuclein levels than CathB [19,20]. Interestingly, in our studies, both proteases were capable of processing mHtt to an equivalent extent. This may be due to the much bigger size of the mHtt protein that allows for more proteolytic cleavage sites compared to α-synuclein. It is interesting to note that endogenous htt levels are not significantly decreased by CathD or CathB transfection. Prior studies demonstrated that endogenous normal htt plays an important role in development [22], although its exact role in neurons has not been well-established. Our observation that increased CathD and CathB only reduce excessive htt, without affecting basal level endogenous htt, is most likely because only in the case of overproduction of htt is the lysosomal system engaged for clearance of this protein. This would be consistent with a potential benefit of increasing these lysosomal activities for reduction of toxic proteins without reducing endogenous functional proteins. mHtt can be digested by proteasomes, caspases, cathepsins and gamma-secretase in a variety of cell models [5,6,27-29]. For example, inhibiting proteasomes by inhibitors such as ALLN and lactacystin was shown to promote the accumulation of NH_2_-terminal Htt fragments in both HEK293 cells and in brain tissues. Moreover, the endogenous NH_2_-terminal mutant Htt fragments accumulated in the brains by proteasome inhibition [5,6,27-29]. The Autophagy-lysosome pathway is a key clearance pathway for mHtt fragments. Induction of autophagy protects against polyglutamine toxicity and inhibition of autophagy has the converse effects [5,6,27-29]. Our observations indicate that *in vitro*, up-regulation of CathD or CathB does not lead to increased smaller mHtt fragments, but reduced overall full length and smaller fragments, consistent with a function of the lysosomal enzymes in complete rather than partial degradation of mHtt.

mHtt was shown to affect protein levels and maturation of CathD in optineurin/Rab8-dependent trafficking pathway from post-Golgi to lysosomes [30]. However, in our studies, CathD and CathB mature enzymes are produced both in the absence and presence of mHtt. And the CathD and CathB enzymatic activities are similar in the absence and presence of mHtt. Furthermore, exogenously expressed CathD and CathB are localized to the lysosomes. There could be a number of reasons for these differences between ours and the prior studies, including: a) different cells and constructs for the expression of mHtt; and b) overexpressed CathD and CathB in our studies reduced the levels of mHtt below the levels needed to block protein trafficking.

145QmHtt over-expression in primary cortical neurons resulted in increased cytotoxicity which was further enhanced by the inhibitors of cathepsin B and D. 3-methyladenine (3-MA), which inhibits autophagosome formation due to its inhibitory effects on the VPS34-Beclin complex, had a similar effect on mHtt toxicity as the cathepsin inhibitors. The fact that the combined effects of cathepsin B and D inhibition were greater than either alone and similar to 3-MA suggest that both proteases make a significant contribution to mHtt degradation.

Prior studies have analyzed mTOR-dependent and independent pathways in stimulating macroautophagy [31]. Enhancing macroautophagy may be beneficial to degradation of mHtt aggregates [2,3,31-34]. Rapamycin induces autophagosome formation and reduces mHtt aggregates, albeit this appears to be a somewhat inefficient process [32]. One explanation is that autophagosomal activity is efficient in healthy neurons, such that accumulation of autophagosomes is a rare event [4]. In contrast, lysosomal protease activities seem to be rate-limiting and easily disturbed as evidenced in a variety of lysosomal diseases [4], and their activities decline with age [10,24,35]. Recent studies also demonstrated that up-regulation of lysosomal biogenesis reduced mHtt levels in cell lines [36], highlighting the importance of the lysosomes. Surprisingly, we did not observe LC3 II/LC3 I conversion increase in CathD and CathB transfected neurons, one explanation may be the autophagy flux is too fast to measure LC3 II accumulation in CathD and CathB transfected neurons. Interestingly, our studies also observed that LC3 II/LC3 I ratio is upregulated in primary neuron cultures transfected with CathD and CathB in the presence of 23QHtt. This observation suggests a feedback regulation by signals from the lysosome to the machinery regulating autophagosomal formation. Recent studies suggest that mTOR may be in a complex on the lysosomal membrane and can serve as a signal for autophagy initiation [37]. Future studies will critically investigate how lysosomal activities influence autophagy initiation through either an mTOR-dependent or an mTOR-independent pathway. In the HdhQ200 knock-in mouse model, mHtt aggregates colocalize with LC3 positive staining, suggesting accumulation of mHtt within uncleared autophagosomes [26]. Consisting with this observation, our studies also showed 145QmHtt transfected neurons had increased LC3 II/LC3 I ratio compared to 23QHtt transfected neurons. CathD or CathB transfected neurons that express 145QmHtt exhibit reduced LC3 II/LC3 I ratio, suggesting CathD and CathB play a role in helping clear the accumulated autophagosomes in these neurons. Future studies will also need to critically investigate whether CathD and CathB can serve not only to attenuate nascent mHtt aggregate formation, but also to enhance clearance of existing pre-formed aggregates [38].

## Conclusions

**Figure 5:**
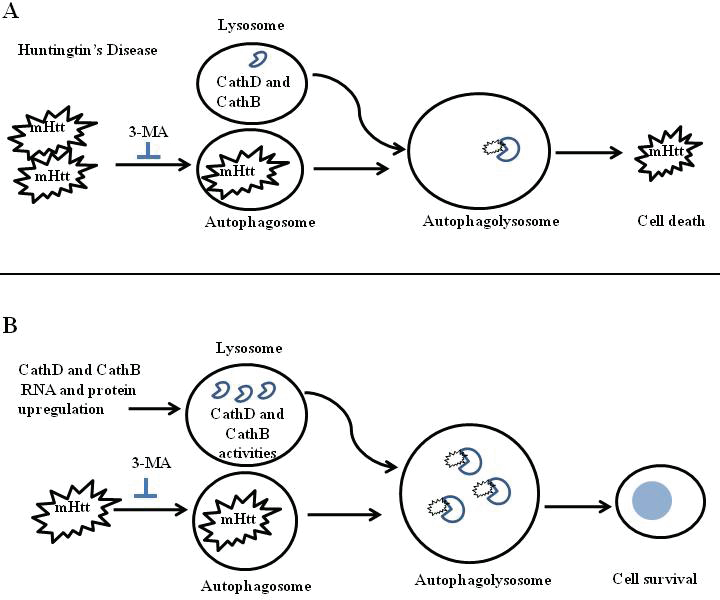
**A model for neuroprotective effects of lysosomal cathepsins D and B against mHtt toxicity**. **A**. In Huntington's disease conditions, mHtt cannot be completely degraded and accumulates, leading to neurotoxicity. 3-MA inhibits autophagy activity and exacerbates mHtt-induced neurotoxicity. **B**. Increasing lysosomal cathepsins D and B reduces mHtt accumulation, and confers neuroprotection. Neuroprotection is dependent on autophagy, since 3-MA reduces cells survival even in the presence of CathD or B.

This study demonstrates that enhanced activity of the individual lysosomal proteases CathD or CathB, reduced 145Q mutant huntingtin level and toxicity in multiple cell models. Neuroprotection by CathD or B is associated with changes and dependent on the macroautophagy pathway. The proposed model for CathD and B neuroprotection against mHtt accumulation and toxicity is shown in Figure 5. These observations lay a foundation for further investigating molecular and cellular mechanisms of autophagy-lysosomal regulation and therapeutic potential of enhancing lysosomal cathepsins in reducing mutant huntingtin accumulation and toxicity.

## List of abbreviations

CathB: cathepsin B; CathD: cathepsin D; DIV: days *in vitro*; Htt: huntingtin; mHtt: mutant huntingtin; 3-MA: 3-methyladenine; pepA: pepstatin A.

## Competing interests

The authors declare that they have no competing interests.

## Authors' contributions

QL performed the majority of the experiments and data analyses. XO and LS assisted with some of the experiments. QL and JZ wrote the manuscript. JZ directed the research. All authors read and approved the final manuscript.

## Supplementary Material

Additional file 1 Additional Figures S1, S2 and S3 (JPEG) Click here for file

Additional file 2 Additional file legends (word) Click here for file
