# Supplementary material for: Reduction of mutant huntingtin accumulation and toxicity by lysosomal cathepsins D and B in neurons
Source: Mol Neurodegener. 2011 Jun 1;6:37. doi: 10.1186/1750-1326-6-37 (PMC3164227; doi:10.1186/1750-1326-6-37)
Supplement: Additional file 1 — Additional Figures S1, S2 and S3 (JPEG) [file 1750-1326-6-37-S1.PPTX]

## Slide 1
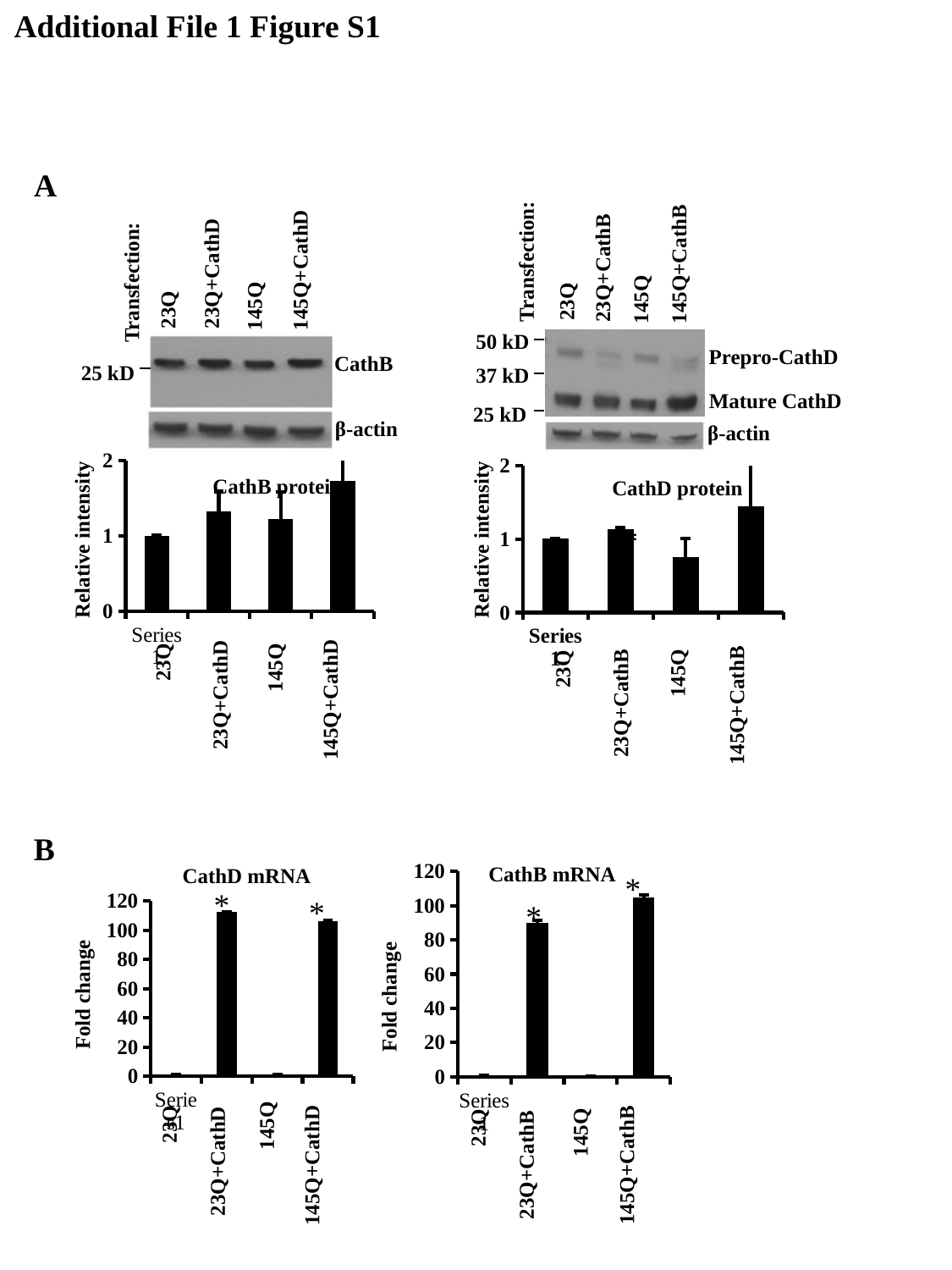

Additional File 1 Figure S1
A
145Q+CathD
23Q+CathD
Transfection:
145Q
23Q
CathB
β-actin
25 kD
145Q+CathB
23Q+CathB
145Q
23Q
Prepro-CathD
Mature CathD
β-actin
50 kD
37 kD
25 kD
### Chart
| Category | CB level |
|---|---|
| | 1.001290999999994 |
| | 1.3257363333333332 |
| | 1.2262616666666666 |
| | 1.7282663333333335 |Relative intensity
145Q+CathD
23Q+CathD
145Q
23Q
CathB protein
### Chart
| Category | CD level |
|---|---|
| | 1.0011233333333334 |
| | 1.1291293333333332 |
| | 0.7489793333333393 |
| | 1.444612 |Relative intensity
CathD protein
145Q+CathB
23Q+CathB
145Q
23Q
Transfection:
B
### Chart
| Category | |
|---|---|
| | 1.0 |
| | 90.03 |
| | 0.599 |
| | 104.72 |CathB mRNA
*
*
145Q+CathB
145Q
23Q
23Q+CathB
CathD mRNA
*
### Chart
| Category | |
|---|---|
| | 1.0 |
| | 112.47 |
| | 1.27 |
| | 106.49000000000002 |*
145Q
145Q+CathD
23Q
23Q+CathD

## Slide 2
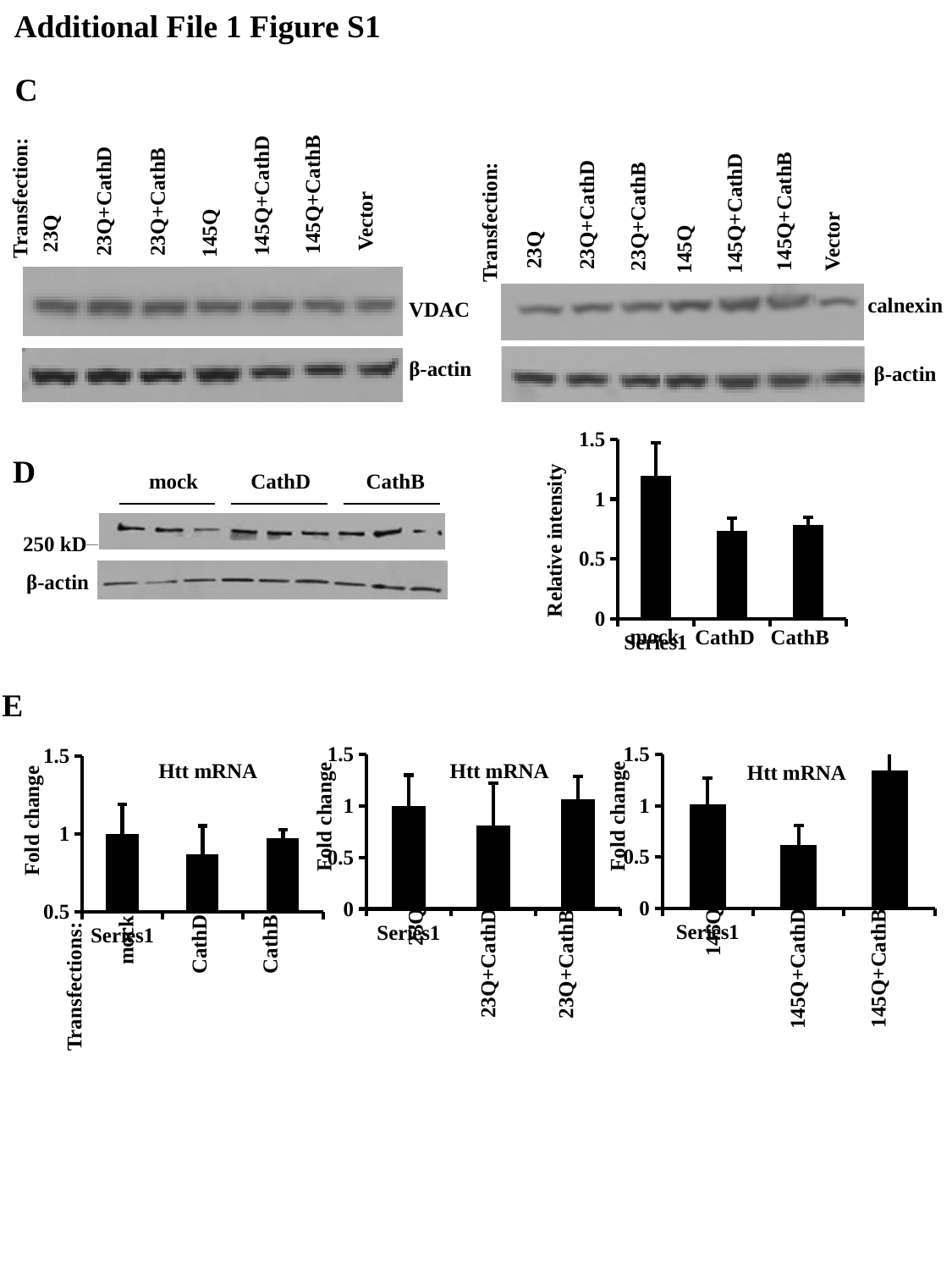

Additional File 1 Figure S1
C
145Q+CathB
145Q+CathD
23Q+CathD
23Q+CathB
Vector
145Q
23Q
VDAC
β-actin
Transfection:
145Q+CathB
145Q+CathD
23Q+CathD
23Q+CathB
Vector
23Q
calnexin
β-actin
145Q
Transfection:
### Chart
| Category | |
|---|---|
| | 1.1959761902569004 |
| | 0.7347545100367546 |
| | 0.7875276895950045 |mock
CathB
CathD
D
mock
CathD
CathB
250 kD
β-actin
E
Fold change
### Chart
| Category | |
|---|---|
| | 1.0000738095238124 |
| | 0.8145226190476186 |
| | 1.067492261904762 |23Q+CathD
23Q
23Q+CathB
Htt mRNA
Fold change
### Chart
| Category | |
|---|---|
| | 1.015970172765353 |
| | 0.6178663474488592 |
| | 1.3417062663291632 |145Q+CathB
145Q
145Q+CathD
Htt mRNA
### Chart
| Category | |
|---|---|
| | 1.0004706257794018 |
| | 0.8720985242582829 |
| | 0.9723172550636602 |CathD
CathB
mock
Fold change
Htt mRNA
Transfections:

## Slide 3
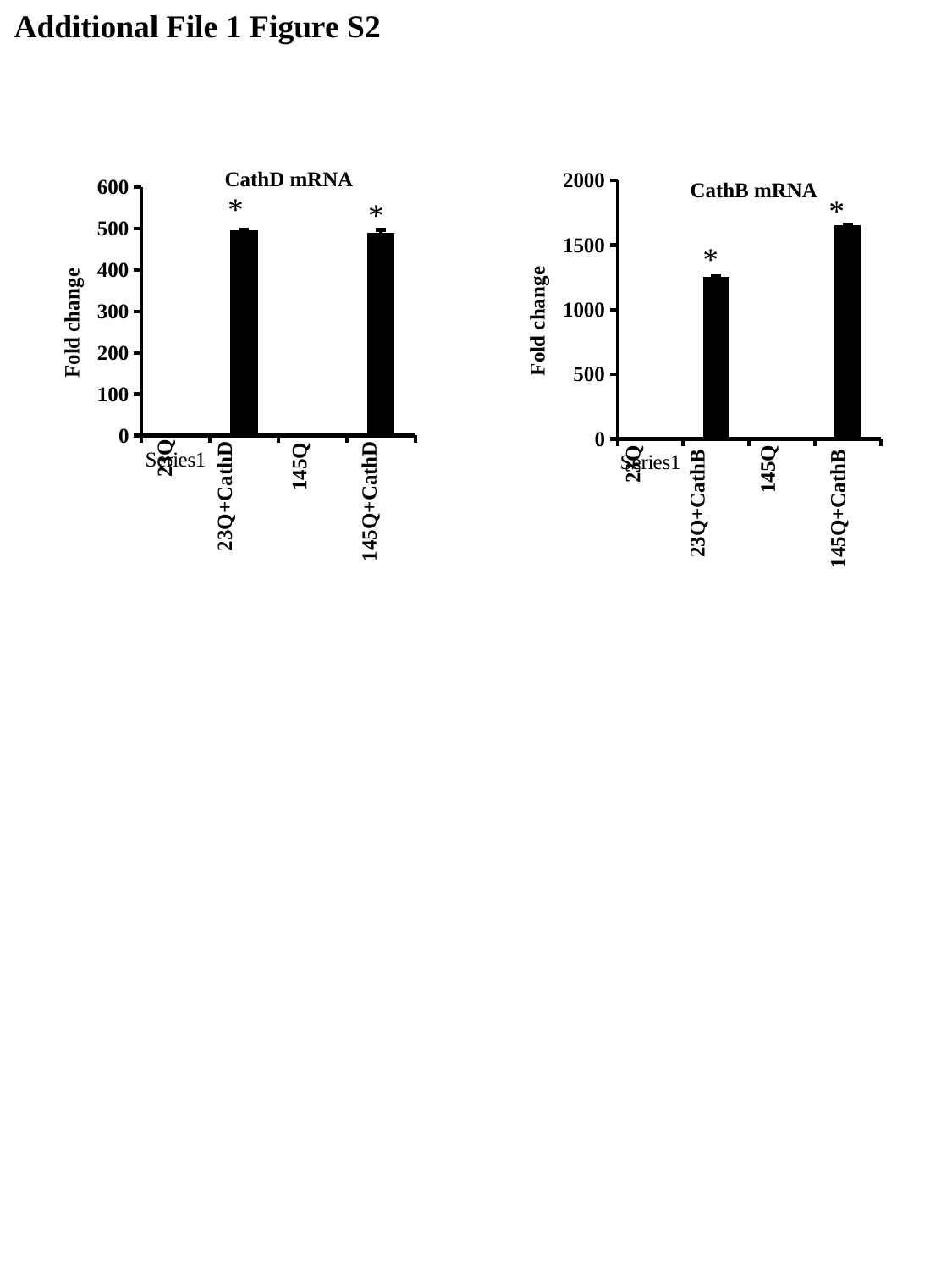

Additional File 1 Figure S2
CathD mRNA
### Chart
| Category | |
|---|---|
| | 1.0 |
| | 495.75 |
| | 1.56 |
| | 489.489999999999 |*
*
23Q
145Q+CathD
23Q+CathD
145Q
### Chart
| Category | |
|---|---|
| | 1.0 |
| | 1256.47 |
| | 0.2400000000000002 |
| | 1656.41 |*
*
145Q
23Q
145Q+CathB
23Q+CathB
CathB mRNA

## Slide 4
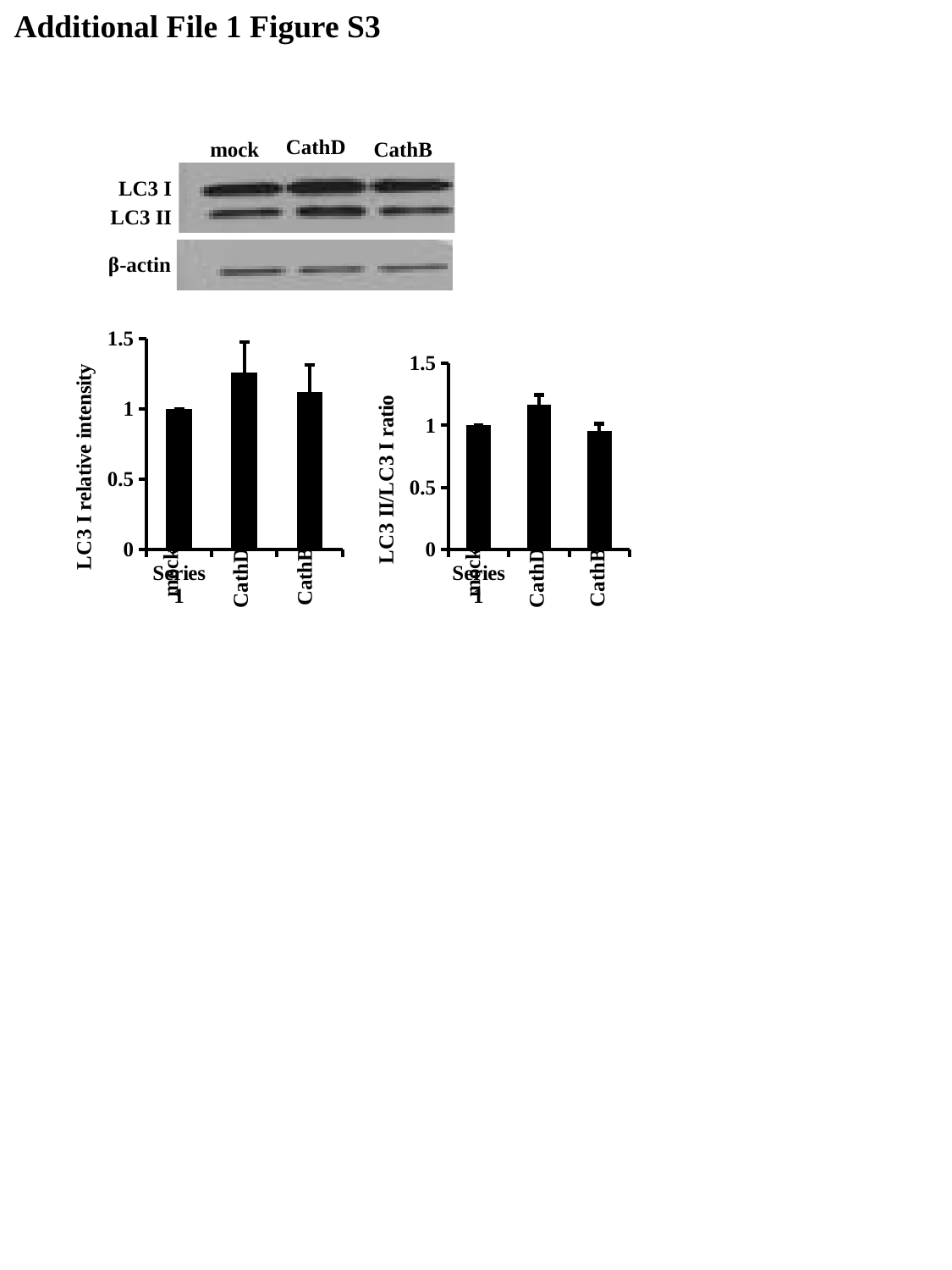

Additional File 1 Figure S3
CathD
mock
CathB
LC3 I
LC3 II
β-actin
### Chart
| Category | |
|---|---|
| | 1.000049 |
| | 1.2583996666666668 |
| | 1.1195156666666681 |mock
CathB
CathD
### Chart
| Category | |
|---|---|
| | 1.0000434999999999 |
| | 1.1690536666666693 |
| | 0.9536230000000012 |mock
CathD
CathB
