# Supplementary material for: Reduction of mutant huntingtin accumulation and toxicity by lysosomal cathepsins D and B in neurons
Source: Mol Neurodegener. 2011 Jun 1;6:37. doi: 10.1186/1750-1326-6-37 (PMC3164227; doi:10.1186/1750-1326-6-37)
Supplement: Additional file 2 — Additional file legends (word) [file 1750-1326-6-37-S2.DOCX]

**Additional file legends:**

**Fig.1. A.** Cathepsin D (CathD) and B (CathB) do not elicit significant cross regulation in HEK cells. HEK cells were transfected with 23QHtt, 23QHtt plus CathD, 23QHtt plus CathB, 145QmHtt, 145QmHtt plus CathD, and 145QmHtt plus CathB constructs for 48 hr. There is a modest induction of CathB by CathD transfection, and a modest induction of mature CathD by CathB transfection. **p* < 0.05 compared to without cathepsin transfection. **B.** Increase of CathD and CathB mRNA after transfection. HEK cells were transfected as in A. CathD and CathB mRNA levels were determined by real-time PCR. Relative quantity of RNA was normalized against without CathD or CathB. **p* < 0.05 compared to without cathepsin transfection.  **C.** Transfection of CathD or CathB does not reduce levels of other intracellular proteins, including mitochondrial outer membrane protein VDAC and endoplasmic reticulum protein calnexin by western blot analyses. Experiments were repeated n=3. **D.** Transfected CathD or CathB did not reduce endogenous Htt. Ab2166 antibody was used to detect endogenous Htt in the western blot analyses. β-actin western blot was used as a loading control. Western blot intensity was plotted in bar graph. **E.** The mRNA levels of Htt were not affected by CathD or CathB transfection as shown by quantitative RT-PCR. Relative levels of Htt mRNA were quantified by real-time RT-PCR after indicated transfection. Each graph was normalized to the mock, 23Q, or 145Q transfected cells.

**Fig. 2.** Real-time RT-PCR analyses of CathD and CathB mRNA after transfection. Primary cortical neurons were transfected with 23QHtt, 23QHtt plus CathD, 23QHtt plus CathB, 145QmHtt, 145QmHtt plus CathD and 145QmHtt plus CathB constructs. CathD and CathB mRNA levels were determined by real-time PCR. **p* < 0.05 compared to without cathepsin transfection.

**Fig.3.** Cathepsin D (CathD) and B (CathB) do not affect LC3II/LC3I ratio. Primary neurons were transfected with CathD or CathB. Western blot analyses were performed with an anti-LC3 antibody. β-actin western blots were used as loading controls. Immunoreactive bands were quantified and shown in the graphs.
